# Biofilm formation: mechanistic insights and therapeutic targets

**DOI:** 10.1186/s43556-023-00164-w

**Published:** 2023-12-15

**Authors:** Xinyu Wang, Ming Liu, Chuanjiang Yu, Jing Li, Xikun Zhou

**Affiliations:** 1 Department of Biotherapy, Cancer Center and State Key Laboratory of Biotherapy, West China Hospital, Sichuan University, Chengdu, 610041 China; 2 State Key Laboratory of Oral Diseases, National Clinical Research Center for Oral Diseases, Chinese Academy of Medical Sciences Research Unit of Oral Carcinogenesis and Management, West China Hospital of Stomatology, Sichuan University, Chengdu, 610041 Sichuan China; 3 Institute for Cancer Genetics, Columbia University, New York, NY 10032 USA

**Keywords:** Biofilm, *Pseudomonas*, Anti-biofilm drugs, Antibiofilm therapeutic strategy

## Abstract

Biofilms are complex multicellular communities formed by bacteria, and their extracellular polymeric substances are observed as surface-attached or non-surface-attached aggregates. Many types of bacterial species found in living hosts or environments can form biofilms. These include pathogenic bacteria such as *Pseudomonas,* which can act as persistent infectious hosts and are responsible for a wide range of chronic diseases as well as the emergence of antibiotic resistance, thereby making them difficult to eliminate. *Pseudomonas aeruginosa* has emerged as a model organism for studying biofilm formation. In addition, other *Pseudomonas* utilize biofilm formation in plant colonization and environmental persistence. Biofilms are effective in aiding bacterial colonization, enhancing bacterial resistance to antimicrobial substances and host immune responses, and facilitating cell‒cell signalling exchanges between community bacteria. The lack of antibiotics targeting biofilms in the drug discovery process indicates the need to design new biofilm inhibitors as antimicrobial drugs using various strategies and targeting different stages of biofilm formation. Growing strategies that have been developed to combat biofilm formation include targeting bacterial enzymes, as well as those involved in the quorum sensing and adhesion pathways. In this review, with *Pseudomonas* as the primary subject of study, we review and discuss the mechanisms of bacterial biofilm formation and current therapeutic approaches, emphasizing the clinical issues associated with biofilm infections and focusing on current and emerging antibiotic biofilm strategies.

## Introduction

The majority of microorganisms do not exist in the planktonic form in the natural environment. They are most often surrounded by self-secreted polymeric extracellular matrix – this state of existence among microorganisms is called biofilm [1, 2]. Biofilms are complex three-dimensional structures composed of various bacteria and fungi. Biofilms are constantly changing environments that exhibit heterogeneity, and they are characterized by dynamic oxygen gradients, fluctuations in nutrient levels and variations in pH. Due to their complex structure, biofilms are more active in responding to external stimuli and are more resistant to antibiotics than planktonic cells, especially conventional antimicrobial agents [3, 4].

These microcosmic communities profoundly affect a wide range of our daily lives. They directly lead to suppurative infection in humans, and they adhere to medical devices. Therefore, they are strongly associated with nosocomial infections, periodontitis, chronic wound changes, musculoskeletal infections (osteomyelitis), native valve endocarditis, and even malfunction of medical devices [5–9]. In addition, biofilms are the dominant growth mode of foodborne pathogens.

Bacterial biofilms are conglomerations of one or more bacterial species adhering to a surface and shielded by a self-generated matrix comprising polysaccharides, proteins, glycoproteins and nucleic acids [10, 11]. Biofilm formation can lead to a variety of complications, including catheter and contact lens colonization, biopsy infections and accompanying persistent inflammation, endocarditis, wounds, and epithelial infections of the lungs, especially in patients with cystic fibrosis. Table 1 provides an overview of key bacterial species renowned for their biofilm-forming capabilities [10] (Table 1).

**Table 1:** Well-studied biofilm-forming pathogenic bacteria

**Mode of action**	**Substrates/supports for biofilm formation**	**Bacteria**	**Ref.**
Persister cells	Urinary tract Urethral catheters	*Escherichia coli*	[12]
AHL molecules Persister cells eDNA	Central venous catheters Ventricular assist devices Endotracheal tubes Coronary stents Cochlear implants	*Pseudomonas aeruginosa*	[13, 14]
Poly-β(1–6)-N-acetylglucosamine (PNAG)	Coronary stents Peritoneal dialysis catheters Cochlear implants	*Staphylococcus aureus*	[15]
Polysaccharide intercellular adhesion (PIA)	Central venous catheters Orthopedic prostheses	*Staphylococcus epidermidis*	[16]
LuxS	Endotracheal tubes Nasopharynx	*Streptococcus pneumonia*	[17]

*Pseudomonas*, particularly *Pseudomonas aeruginosa* (*P. aeruginosa*), is one of the most common causes of nosocomial infections; these infections mainly manifest as pneumonia, suppuration of exposed wounds, and bacteraemia, and *Pseudomonas* is second only to *Staphylococcus aureus* (*S. aureus*) in terms of causing nosocomial infection [18, 19]. The respiratory tract is a common habitat of *P. aeruginosa*, in which microorganisms are distributed nonrandomly and take the form of biofilms [20]. The complex architecture of the *P. aeruginosa* biofilm represents an additional determinant in the pathogenicity of this microorganism. This complexity not only contributes to therapeutic challenges but also facilitates evasion of the immune system and the establishment of chronic infections that prove resistant to eradication [21–23]. Therefore, it is of vital significance to summarize the detailed investigations into the composition, intramembrane ecology, regulatory mechanisms and dispersion mechanisms of biofilm formation in *Pseudomonas*.

## Overview and formation process of biofilms

**Fig. 1:**
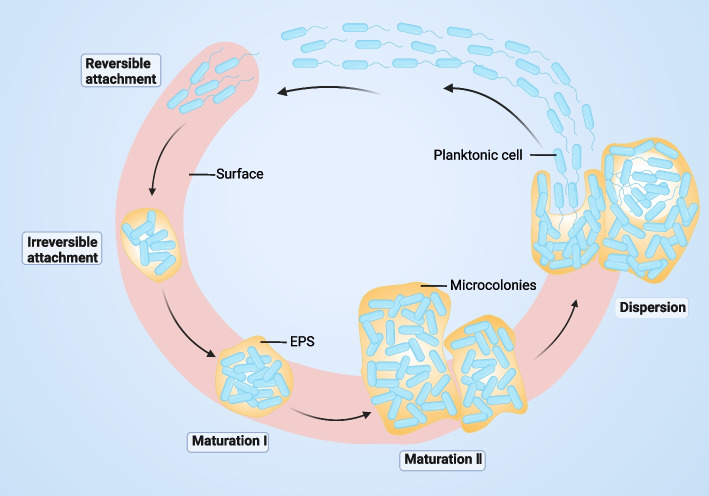
Bacterial biofilm development produces a classic structure. The formation of biofilms consists of five stages: reversible attachment, irreversible attachment, maturation stage I, maturation stage II and dispersion. In the reversible attachment stage, bacteria attach to the matrix through the swing of flagella. In the irreversible attachment stage, the expression of the flagella gene is lost. Then, several cell clusters mature, and thick cell clusters embedded in the biofilm matrix enter mature stage I. In mature stage II, the bacterial clusters reach the maximum thickness, and microcolonies can be seen. When the biofilm is dispersed, the biofilm cycle will cycle again

Biofilms are a natural form of growth for most microorganisms, such as bacteria and mycoplasma fungi, etc. Biofilm development is a rapid process and usually needs to go through several stages, including reversible attachment, irreversible attachment, biofilm maturation and, finally, dispersion. These stages of biofilm development are connected with changes in cell phenotype. These stages are connected with changes in bacterial biological characteristics such as nutrition metabolism and growth speed, and the most important is the change in drug resistance (Fig. 1).

### Adhesion

Regarding biofilm formation, taking *P. aeruginosa* as an example, biofilm development occurs in five stages: reversible attachment, irreversible attachment, maturation-1, maturation-2, and dispersion [24, 25]. In a liquid culture suspension, *P. aeruginosa* could survive in a planktonic state. However, on natural or synthetic surfaces, *P. aeruginosa* attaches to the substratum by means of their extracellular appendages, such as flagella and pili (facilitating swimming and twitching motility), reversibly or irreversibly [26, 27]. Surface-attached communities significantly contribute to their persistence and drug resistance, and microorganisms that inhabit biofilms usually exhibit greater heterogeneity and more differences in phenotype and metabolic activity compared with the same strains of the planktonic form [28, 29]. In the reversible attachment stage, the fragile and weak adhesive force between planktonic *P. aeruginosa* cells and the substrate is composed of van der Waals forces and hydrophobic interactions. Once temporary attachment is established, *P. aeruginosa* strains stretch their type IV pili, which mediates irreversible cell-to-surface colonization [30]. Relatively irreversible covalent and hydrogen bonding interactions contribute to irreversible attachment, whereas nonspecific interactions are dominant in attachment onto abiotic surfaces [31]. Cyclic dimeric guanosine monophosphate, known as cyclic di-guanosine monophosphate (c-di-GMP), plays a vital role in the regulation of biofilm formation, motility, adhesion, virulence and morphogenesis. The basal concentration of c-di-GMP promotes dispersal and structures, and a high cellular concentration of c-di-GMP enhances matrix component production, including exopolysaccharides [32].

### Attached component

Psl (polysaccharide synthesis locus) is a neutral repeating pentasaccharide that is the key component of biofilm adhesion. According to studies focusing on the biophysical and biomechanical mechanisms, Pel (pellicle polysaccharide) provides transient adhesive force that is not as permanent as Psl. Type IV pili are enriched in Psl-rich regions, and a high level of Psl generates short-range and localized attachment by paralleling rod-shaped *P. aeruginosa* to the substrate [24, 33]. Extracellular DNA (eDNA) is another major accelerant of early biofilm development that facilitates motility-mediated expansion. In contact with Pel and Psl, eDNA promotes the stability of the biofilm, and eDNA-deficient biofilms usually exhibit higher sensitivity to detergents [34, 35]. Strong biofilm producers often accumulate higher amounts of exopolymeric substances, including eDNA, protein, and pel polysaccharide [36]. In addition, CdrA acts as an extracellular adhesin that is secreted with the CdrA-CdrB two-partner secretion system [37]. It is regulated by c-di-GMP; typically, a low level of c-di-GMP helps more CdrA transport out of the cell [38, 39]. By interacting with Pel and Psl, CdrA maintains the integrity of the biofilm structure [40]. In addition, even without exopolysaccharides, it can also promote cell clustering [24].

Studies investigating bacterial adhesion and biofilm formation on biomaterial mechanical properties and growth medium found that magnesium, instead of calcium, determines the aggregation of dense *P. aeruginosa* and high levels of c-di-GMP, which is contrary to previous studies [32]. It was discovered that intracellular c-di-GMP levels are also regulated by the Wsp chemosensory-like signal transduction pathway, which could be activated by the methyl-accepting chemotaxis protein WspA [41]. The receptors for c-di-GMP promoted by WspR are FleQ and PelD [42, 43]. FleQ increases exopolysaccharide expression by activating the corresponding operon, further inhibiting flagellum gene expression, eliminating surface sensing and promoting irreversible attachment [44, 45].

For humans, *P. aeruginosa* biofilms can form within compromised respiratory epithelium, particularly in individuals with chronic lung disease or those undergoing mechanical ventilation with infections [46]. Bacteria adhere to the respiratory epithelium via type IV pili and flagellum, subsequently secreting extracellular matrix to form biofilms and release toxins that damage lung tissue [47, 48]. Some bacteria may break free and spread the infection. In patients with chronic lung diseases such as CF or chronic bronchiectasis, biofilm development can take place within the lung tissues. *P. aeruginosa* organisms have the capability to form biofilms within thickened airway mucus, often without the necessity to migrate to cell surfaces [49]. The loss of flagellar motility could hinder the surface attachment of *P. aeruginosa*; however, the loss of flagellar motility could still form nonattached aggregates that share characteristics with biofilms, including increased antibiotic tolerance. These studies expand the conceptual model of biofilm formation, which can occur in both surface and non-surface environments [50].

### Plaque maturation

When irreversibly attached, the motile cells continue to pour into the biofilm initiation, forming microcolonies. Subsequently, biofilms begin to express large amounts of extracellular polymers and exopolysaccharides, mainly represented by Pel, Psl and alginate, i.e., the central building blocks [51, 52]. The initiation of biofilm maturation is characterized by cell proliferation and the loss of motility organelles. When the biofilm is mature and thickened, the three-dimensional structure of the biofilm gradually forms, thus turning into a mushroom-shaped multicellular assembly [53]. C-di-GMP signalling maintains biofilm-needed YfiN to limit motility and protect viability in response to peroxide stress [54, 55].

Intracellular and extracellular polysaccharides not only give rise to biofilm structural stability but also play a vital role in resisting host immunity and internal and external environmental stimuli, as well as maintaining antibiotic resistance. In a mouse model that lacks Pel and Psl, the size and spatial distribution of biofilms in wound tissue greatly differ from those in the control group, and the ability to survive antibiotic treatment weakens [51]. Alginate, a type of extracellular polysaccharide, is widely accumulated in the biofilm of the *P. aeruginosa* mucous strain, which is important for its maturation and stability [56]. Alginate production is limited by the anti-sigma factor MucA, while in the airways of individuals with chronic lung infections, a mutation resulting in uninhibited alginate production is acquired [57–59].

## Composition and intramembrane ecology of biofilms

**Fig. 2:**
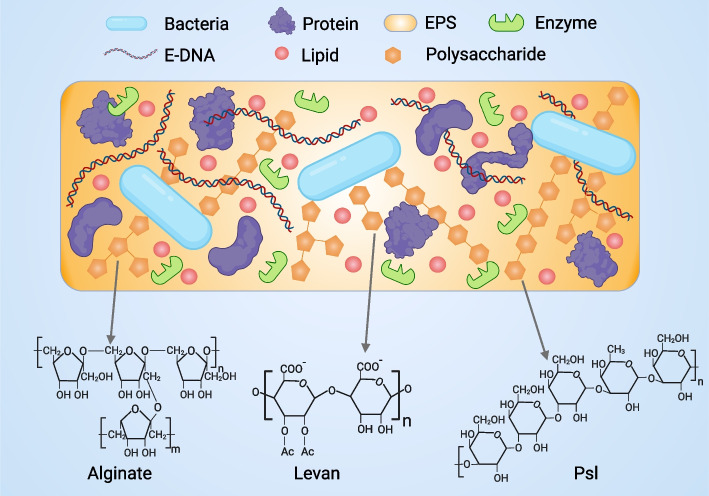
Abundant biofilm matrix molecules. In the matrix of biofilms, polysaccharides are important components, including alginate, fructan and other capsular polysaccharides, as well as Psl and other aggregation polysaccharides. Alginate is a highly molecular-weight acetylated polymer and is an anionic polysaccharide composed of β-1-4 glycosidic bond-linked α-α L-guluronic acid and β-D-mann β uronic acid. Levan is a high molecular weight b-2,6 polyfructose with extensive branching through the b-2,1 bond. Psl polysaccharides are neutral polysaccharides consisting of pentasaccharide repeating units composed of D-mannose, D-glucose, and L-rhamnose. In addition, there are a large number of proteins, enzymes, eDNA, lipids and other important components in the matrix

Bacteria produce extracellular polysaccharides, nucleic acids and protein matrices by wrapping around themselves to form a multicellular structure. Biofilm-producing bacteria depend on extracellular polymeric substances (EPS), also referred to as the matrix, which play a vital role in enabling both surface and volume colonization [59] (Fig. 2). Mature biofilms contain almost 5-25% bacterial cells and 75-95% extracellular polymeric matrix [60]. Extracellular polymers are components of biofilms with thicknesses ranging from 0.2 to 1.0 μm and have been described in both gram-positive and gram-negative bacteria. Generally, biofilms take the form of dense beds consisting of a viscous mixture of polysaccharides, other polymers, and water. EPS use electrostatic, van der Waals, and hydrogen bonding forces to adhere biofilms to the surface of solids and to promote maturation of the biofilm. In some mature biofilms, the water-filled channels make them similar to the original multicellular organisms.

In the matrix of biofilms, polysaccharides are important components, including alginate, fructan and other capsular polysaccharides, as well as Psl and other aggregation polysaccharides. In addition, there are a large number of proteins, enzymes, eDNA, lipids and other important components in the matrix. In the process of biofilm formation, exopolysaccharides, nucleic acids and proteins are the main components of biofilms. Different types of EPS can affect the final biofilm morphology, such as mucinous biofilms, mushroom-like biofilms, and filamentous biofilms [61]. In most cases, at a particular stage of biofilm formation or in a single strain, one or two of these constituents tend to be most copious within the biofilm matrix, although it is common for other components to have auxiliary functions [62]. Primarily, bacteria require polysaccharides in their biofilm matrix at several stages of development, while nucleic acids are utilized in the later stages of maturation. Based on their roles within microbial habitats, i.e., niche biology, the functionality of these biofilm matrix components should also be examined, i.e., niche biology [59].

### Polysaccharides

As the most important matrix of biofilm components, exopolysaccharides have been shown to affect the final biofilm morphology [63]. Exopolysaccharide components are skeletal components of biofilm support structures that provide nutrients for bacteria and increase structural stability. Two different types of polysaccharides – capsular polysaccharides and aggregated polysaccharides – have been demonstrated to contribute to the process of biofilm formation [64]. Capsular polysaccharides retain the properties of a protective dynamic polymer, serving as an external adornment for one or more cells. Concurrently, aggregated polysaccharides offer structural support and engage in interactions with other matrix components [65]. Capsular polysaccharides form a protective coating around the bacteria, whereas aggregating polysaccharides do not.

#### Capsular polysaccharides

Capsular polysaccharides are divided into two categories: alginate and levan. In studies of *P. aeruginosa*, the alginate produced by this species was significantly correlated with adverse clinical outcomes [61]. Investigations in other *Pseudomonas* species have shown that they produce alginates similar to those produced by *P. aeruginosa* [64]. Furthermore, levan produced by different Pseudomonas strains exhibits structural and compositional distinctions from alginate, suggesting its possession of distinctive functions distinct from those of alginate [62].

*Capsular polysaccharides:* Alginate is a high molecular weight acetylated polymer and is an anionic polysaccharide composed of β-1-4 glycosidic bond-linked α-α L-guluronic acid and β-D-mann β uronic acid [66]. Typically, it undergoes O-acetylation at the 2 and/or 3 positions on D-mannic acid residues. B-1,4 and B-1,3 connections are generally quite rigid compared to the b-1,2 and b-1,6 connections commonly found in dextrose esters. Alginate and biosynthesis are well conserved in *Pseudomonas* [67].

Mucinous biofilms induced by the overproduction of alginate occur mainly when *P. aeruginosa* infects the body [68]. Infection of the lungs in cystic fibrosis causes inflammatory cells to be transferred to the site of infection, and the released reactive oxygen species cause extensive tissue damage. The excessive production of alginate by *P. aeruginosa* can protect itself from this inflammatory response. Alginate can scavenge free radicals released by the body and protect bacteria from phagocytosis clearance by activated macrophages. In addition, excessive production of alginate by *P. aeruginosa* can reduce its own synthesis of some virulence factors, such as siderophores and rhamnolipids, during infection. Therefore, the host's immune response is weakened, and coinfection with other bacteria, such as *S. aureus*, is promoted [62, 67, 69].

Alginate makes colonies appear viscous compared to nonmucinous strains and contributes to the structural stability of biofilms. Beyond their fundamental structural roles, biofilm matrix components serve supplementary functions. Capsular polysaccharides such as alginate and levan possess the capacity to retain moisture and nutrients [70]. While the water retention properties of alginate have not been extensively explored, they are anticipated to resemble those of polysaccharides such as hyaluronic acid. Biofilms containing alginate demonstrate a distinct capacity to thrive in challenging environments, which is particularly crucial in bacterial soil life cycles wherein resilience to drought stress plays a pivotal role.

The role of alginate in promoting persistence and immune evasion has been elucidated. Studies have shown that alginates produced by slime strains can make antimicrobials and conditioning phagocytosis unbearable. Alginate also possesses the capability to scavenge free radicals released outside of the body by neutrophils and activated macrophages, which are normally capable of killing bacteria. The pathogenic adaptability of *P. aeruginosa* plays a key role in shaping how polysaccharides, such as alginate, affect its ability to persist and evade the immune system [66]. Alginate, alongside other virulence factors that are present and participate in active infections, contributes to the ability of *P. aeruginosa* to persist in the presence of immune mediators and chronic inflammatory conditions.

In summary, it is evident that alginate production under biofilm-related conditions plays a significant role in environmental persistence and pathogenic adaptation. A more detailed investigation of the underlying mechanisms could provide valuable insights into the biofilm-mediated capabilities of *P. aeruginosa*.

*Capsular polysaccharides: levan* The podophyllotoxin levan is synthesized by a subset of *Pseudomonas* bacteria, especially the plant pathogen *P. syringae*. Levan is characterized by its high molecular weight, consisting of β-2,6-linked polyfructose with widespread branching through β-2,1 bonds [59]. Levan is synthesized from sucrose merely, catalyzed by extracellular levan sucrase. The production of levan is regulated by lad, which is homologous to the lad locus that produces the sensory kinase in *P. aeruginosa*. The specific details regarding levan production and the expression of levan sucrase have yet to be fully elucidated, but it appears that sensing external stimuli plays a critical role [65].

#### Aggregative polysaccharides

Aggregated polysaccharides are alternative substances for biofilm formation in nonmucilaginous strains in the absence of alginate [71, 72]. Matsukawa and Greenberg identified three different loci within *P. aeruginosa* PAO1, which were determined to have the potential to produce components of the polysaccharide matrix. Among these loci, only one, referred to as psl, was found to significantly contribute to biofilm integrity. Disruptions within the other two identified loci resulted in adherence and biofilm formation experiments similar to those of the parental strain [62]. A screening of a transposon insertion mutant library of *P. aeruginosa* PA14 identified that the pel genes are responsible for the production of a glucose-rich matrix material needed for the formation of biofilms [73]. Subsequent studies on nonmucoid biofilms, building upon these reports, have centered their attention on the significance of psl and pel in the process of biofilm formation. In addition, there are also polysaccharide functional alternatives, such as where cellulose appears to substitute for the Pel-related functions of *P. aeruginosa* in pellicle or A–L biofilm formation [61].

*Aggregative polysaccharides: Psl Psl* polysaccharides are neutral polysaccharides consisting of pentasaccharide repeating units composed of D-mannose, D-glucose, and L-rhamnose [59]. Psl is typically found in a minimum of two distinct forms: a high molecular weight component associated with cells and a relatively small soluble form that can be extracted from cell-free culture supernatants. Manipulators (PA2231-PA2245) consisting of *pslA*, *pslB, pslC*, *pslD*, *pslE*, *pslF*, *pslG*, *pslH*, *pslH*, *pslI*, *pslJ*, *pslK*, *pslL*, *pslM*, *pslN*, and *pslO* in the genome of *P. aeruginosa* are responsible for the encoding synthesis of PslD. Recent studies have shown that interactions between PslE and PslA, PslD and PslG facilitate the localization of PslD at the extracellular membrane and the secretion of Psl polysaccharides into the extracellular compartment [62]. Psl polysaccharides are present in two forms within the *P. aeruginosa* biofilm, with the larger oligosaccharide repeating units distributed in a helical fashion on the cell surface, thus playing a role in cell‒cell and cell-substrate surface interactions, and the smaller molecular weight ones distributed in a soluble state throughout the biofilm matrix [74].

Psl polysaccharides are often the first line of defense of bacterial communities within biofilms against antibiotic attack and can resist the toxicity of antimicrobial substances such as cationic antimicrobial peptides (polymyxin B), tobramycin, and ciprofloxacin to *P. aeruginosa* [72]. However, this protective effect of Psl polysaccharides was observed at the early stage of biofilm formation, and the resistance to antimicrobial substances did not increase as the biofilm continued to develop into a mature mushroom-shaped biofilm [66]. In the early stage of biofilm formation, planktonic bacteria showed both fast- and slow-attachment phenotypes when attached to solid surfaces, and this unique attachment phenotype was mediated by the differential expression of Psl polysaccharide produced by the bacteria, which suggests that the differential expression of Psl polysaccharide plays an important role in the development and formation of biofilms at the early stage of biofilm formation [63]. Studies have shown that in the presence of antibiofilm drugs, Psl-producing strains are still able to effectively form biofilms compared to strains that are not able to produce Psl polysaccharides. This suggested that Psl polysaccharides are important for stabilizing the structure of the biofilm and that the immune effects of the abundance of Psl in biofilms may be enhanced [74].

In addition, recent investigations have illuminated the significance of Psl, particularly in the context of the rugose small colony variant (RSCV) of *P. aeruginosa*, suggesting that the RSCV phenotype may confer specific advantages within biofilms or even a competitive edge. Research focusing on the individual effects of enhanced Psl or Pel expression has demonstrated that the overexpression of either polysaccharide, with Psl in particular, results in aggregation and the formation of biofilms [62, 69]. Psl may also play a crucial role in facilitating comparable biofilm adhesion when colonizing immunocompromised patients [75]. Psl regulators are also conserved in pseudomonads other than *P. aeruginosa*. *P. aeruginosa* is limited in biofilm formation in the absence of Psl components, and it may rely on supplementary matrix components to compensate. Notably, Psl is not a prerequisite for biofilm production in *P. aeruginosa* PA14, as this strain lacks the pslABCD gene [59].

*Aggregative polysaccharides: Pel* The gene for the synthesis of the Pel polysaccharide was initially mutated in *P. aeruginosa* PA14 using transposon insertion mutagenesis, and screening of biofilm-deficient strains at the air-liquid interface led to the identification of the manipulator responsible for the synthesis of Pel polysaccharides, PA3058-PA3064, which consists of seven adjacent genes: pelA, pelB, pelC, pelD, pelE, pelF, and pelG [73]. Pel polysaccharides were identified in recent studies as being composed of partially deacetylated N-acetyl-D-glucosamine and N-acetyl-D-galactosamine [76]. Pel polysaccharides are synthesized by the inner membrane complex composed of PelD, PelE, PelF, and PelG and are partially deacetylated in the periplasmic space under the action of PelA deacetylase and become positively charged; then, they are attracted by PelC, a negatively charged outer membrane lipoprotein, and guided to be secreted to the extracellular space by PelB, a pore protein of the outer membrane. It is then attracted by the negatively charged outer membrane lipoprotein PelC protein and guided through the outer membrane pore protein PelB to be secreted into the extracellular compartment, where it protects the bacteria from being killed by aminoglycoside antibiotics [77]. Pel has a clearer effect on biofilm formation in the absence or disruption of the psl manipulator, such as PA14, or when c-di-GMP is maximized. Yang et al. recently found that PAO1 biofilms utilize Pel in the presence of a glycoside hydrolyzing enzyme [78]. It has been recently found that PAO1 biofilms utilize Pel for greater structural stability in microcolony formation. Pel has the ability to reduce antibiotic efficacy during biofilm formation. While biofilm adhesion and overall structural integrity are hallmarks of the contributions made by polysaccharides to biofilm function, recent attention has shifted toward the role of polysaccharides in conferring resistance to antibiotics and influencing cell signalling [59]. In the absence or disruption of the Psl manipulator, as in PA14, or when c-di-GMP is maximized, Pel's impact on biofilm formation becomes more pronounced. Yang et al. recently discovered that PAO1 biofilms rely on Pel for enhanced structural stability in microcolony development. Pel reduces the efficiency of antibiotics during biofilm formation [38]. While biofilm adhesion and overall structural stability are hallmarks of the contribution of polysaccharides to biofilm function, polysaccharide-mediated tolerance to antibiotics and even cellular signalling have recently been emphasized. Polysaccharides can also influence the expression of population sensing and ultimately quinolone *Pseudomonas* quinolone signal (PQS)-mediated eDNA release [79, 80].

### Nucleic acids

Another common and abundant building block of the biofilm matrix is nucleic acids [59]. In particular, DNA serves as a pivotal element within the biofilm matrix, contributing to the initial stages of biofilm formation. However, it represents just one facet of the multifaceted biofilm matrix that develops at later stages of the biofilm process. DNA is located in specific regions of the biofilm rather than throughout the entire region of the biofilm [38]. The spatial and temporal accumulation of DNA and the production of polysaccharides throughout the biofilm formation process indicate that there is a promotion of cellular organization in the biofilm [81]. Identification of the mechanisms of molecular and cellular interactions in the biofilm matrix will aid in the development of therapies for the dispersal and clearance of biofilm infections.

### Polysaccharide-containing matrix components

Beyond the primary polysaccharides, additional components within the biofilm matrix have been identified to contribute to its functionality. Although cyclic β-glucan, lipopolysaccharides (LPS), and membrane vesicles (MV) have not been extensively investigated in the context of *P. aeruginosa*, there is evidence of their association with the biofilm matrix [59].

The significance of LPS as a molecule within the biofilm matrix, or its function on the adhesive surface of *P. aeruginosa*, remains to be fully elucidated. Nevertheless, limited studies have offered valuable insights into the role of lipopolysaccharides in enhancing biofilm stability [62–64]. Initially, LPS garnered attention due to its capacity to influence cell surface charge and relative hydrophobicity. Moreover, LPS has been associated with alterations in attachment, a shift toward adherent growth, and changes in colony morphology in various bacterial species. In essence, *P. fluorescens* WS variants depend on LPS interactions to bolster overall biofilm integrity [82]. Sustained exploration into the role of LPS within biofilm matrices is essential for achieving a comprehensive understanding of the diverse range of biofilm compositions.

MVs are versatile bilayer structures originating from the outer membrane of gram-negative bacteria [83]. Although MVs are abundant in biofilms and have obvious matrix-related functions, further studies will shed more light on the effects of MVs on *P. aeruginosa* biofilms. Evidence points to the involvement of the Las quorum-sensing system in governing the early stages of MV formation during biofilm development. Moreover, it is evident that PQS plays a vital role in MV formation, signifying that the Las quorum-sensing system primarily regulates MVs through PQS activation [79]. Further studies of quorum sensing and MV production will provide greater insights into how biofilm-forming organisms use MVs.

### Protein components of the biofilm matrix

Proteins present in biofilm matrices promote functions including surface adhesion, interactions with other matrix molecules, and matrix stabilization [62]. Proteins such as CdrA found in *P. aeruginosa* possess carbohydrate-binding capabilities, rendering them intriguing candidates for mediating interactions with matrix molecules.

Cellular appendages are also needed for biofilm formation in *P. aeruginosa*, although they are not usually recognized as classical biofilm matrix molecules [65, 84]. Flagella and type IV hyphae assist in biofilm formation during biofilm maturation, and microcolonies are formed by clonal expansion of quiescent cells that form *P. aeruginosa*. The myxoid stalk produces cytotoxic lectins that bind carbohydrates and contribute to biofilm stabilization and structure formation [59]. Other protein-based matrix molecules are continually being discovered as essential elements of biofilm development. Notably, a functional amyloid protein was identified in a strain of *P. fluorescens*. Overexpression of the gene cluster responsible for amyloid production, known as fapA-F, resulted in a substantial enhancement of biofilm adhesion [84].

The principal function of matrix components lies in furnishing structural stability to the biofilm, facilitating cell‒cell interactions and the formation of a framework that enhances nutrient accessibility [74]. In addition to providing a barrier to penetration, other matrix molecules do not directly contribute to the resistance of cell populations containing biofilms, but future research will investigate the nature of biofilm-mediated resistance to antimicrobial drugs [85].

### Rhamnolipids

Rhamnolipids are glycolipid biosurfactants with surface-active properties that are synthesized by various bacteria. However, they were initially discovered in *P. aeruginosa* [59]. Surface adhesion secreted by rhamnolipids is particularly important in environments where nutrient sources are low, helping the species to adapt to a more arduous lifestyle [63, 64]. *Pseudomonas* exhibits remarkable adaptability to environmental stressors via biofilm formation. Rhamnolipids contribute to this adaptability by enhancing surface activity, wetting capability, removal rates, and other amphipathic-related features. The precise mechanism by which *P. aeruginosa* harnesses these high molecular weight hydrophobic compounds as an energy source remains uncertain, and it is plausible that rhamnolipid production plays a pivotal role in this process. In addition to *P. aeruginosa*, many other *Pseudomonas* species also produce rhamnolipids, but their specific functions are not well defined [59].

## Regulatory mechanisms of biofilm formation

**Fig. 3:**
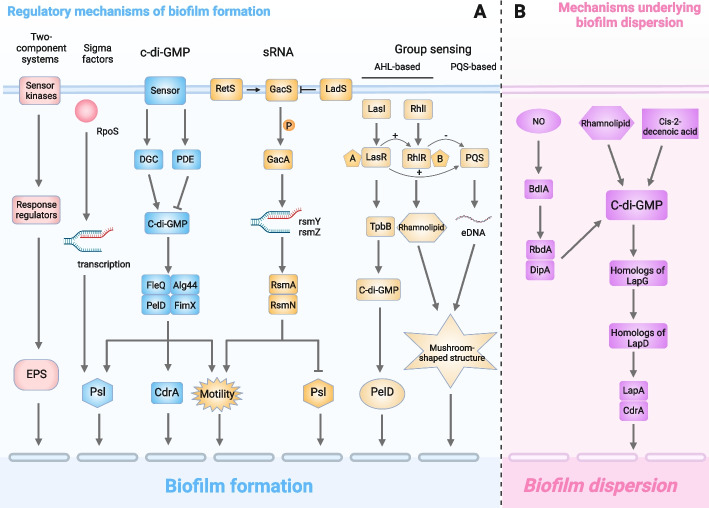
Biofilm formation and dispersion. **a** In the mechanism of biofilm formation, the two-component system is usually composed of sensor kinases and response regulators. Quorum sensing includes AHL-based quorum sensing systems and PQS-based quorum sensing systems. Among them, LasR can positively regulate RhlR and PQS, while RhlR can negatively regulate PQS. LasR can regulate the activity of TpbB, reduce the level of c-di-GMP, and promote biofilm formation by activating PelD. RhlR controls the synthesis of rhamnolipid and maintains the channel in the mushroom-like structure together with eDNA released from the PQS system. The synthesis and degradation of c-di-GMP occurs through the opposite activity of diguanosine cyclase (DGC) with a GGDEF domain and phosphodiesterase (PDE) with an EAL or HD-GYP domain. DGC can promote the synthesis of c-di-GMP, PDE can reduce the level of c-di-GMP, and c-di-GMP will act on the four effectors Alg44, FleQ, PelD and FimX. The formation of biofilms is promoted by regulating the synthesis of Psl and CdrA and convulsive movement. In the sRNA pathway, sensor kinases include GacS, RetS, and LadS; GacS can phosphorylate GacA, and GacA can activate the transcription of rsmZ and rsmY, thereby regulating the synthesis of Psl and the corresponding movement. Sigma factors such as RpoS can positively regulate the expression of Psl to promote biofilm formation. **b** In the mechanism of biofilm dispersion, NO can activate BdlA, and then activated BdlA can recruit and activate RpdA and DipA, which can reduce the level of c-di-GMP. In addition, rhamnolipid and cis-2-decenoic acid can also reduce the level of c-di-GMP, which can increase the activity of the LapG homolog and LapD homolog, resulting in the release of LapA and CdrA and eventually leading to the dispersion of biofilms

The regulatory mechanism of biofilm formation has always been a popular area of research, and we will visualize the role of the regulatory mechanism through the demonstration of pictures [49] (Fig. 3a). The active participation of microbial cells is essential for the adhesion process. EPS has been confirmed as an essential component for the construction of biofilm structures [61]. This formation process requires significant changes in cellular physiology leading to the biofilm phenotype. The controlling mechanism under these changes is essentially chemical signalling, which in the case of biofilm formation is usually based on the existing principle of Ram's induction. The best-studied *P. aeruginosa* has three interconnected QS systems [59]. Two use acyl homoserine lactones (AHLs), and the third uses quinolone signalling molecules. Shedding of individual cells or whole biofilms can be induced by environmental conditions (shear, chemicals, etc.) or can be controlled by the microbial population alone.

The regulatory mechanisms involved in biofilm formation have been gradually revealed recently. It has been shown that a two-component regulatory system, quorum-sensing, cyclic di-GMP signalling and extracellular plasma membrane functional signalling pathway can be involved in the control of biofilm processes [49]. The above four mechanisms can be summarized as follows: (1) promoting the production of EPS and eDNA to facilitate adhesion; (2) enhancing intercellular communication to increase the strength of the biofilm; and (3) regulating the speed of its movement and controlling the initial colonization of the biofilm and the formation of aggregates at the later stage of the biofilm.

The two-component signal transduction system (TCS) is an important signal transduction mechanism in which bacteria can sense, respond and adapt to various environmental changes and then regulate a variety of physiological and biochemical processes of bacteria to adapt to changes in the external environment [86]. The TCS integrates and coordinates various input stimuli and controls the formation of the biological periplasm through various mechanisms, such as cross-regulation [87]. It consists of a receptor histidine kinase (HK) and a homologous response regulator (RR) [88]. Once an external stimulus is recognized, HK is activated, while conserved histidine residues are autophosphorylated. Then, the phosphate group was transferred to the conserved aspartic acid residue of the coupled RR. WalK is a membrane-linked HK of the WalK/WalR TCS that regulates genes involved in cell wall metabolism, biofilm production, virulence regulation, oxidative stress resistance, and antibiotic resistance in low-G+C gram-positive bacteria, including *Enterococcus faecalis* (*E. faecalis*) [89]. Based on RT‒qPCR results, *Bacillus subtilis (B. subtilis)* natto supernatant blocked the peptidoglycan biosynthesis process and the WalK/WalR and bgsA two-component systems of *E. faecalis*, thus inhibiting biofilm formation [90].

Bacteria are capable of autonomously generating and releasing population density-dependent signaling molecules to regulate their community behavior. This regulatory system is known as QS [91]. QS is an important mechanism for intercellular communication, cell adhesion and biofilm formation. Signalling elements are inherently automatic inducers of bacterial synthesis and secretion. The QS regulatory mechanism is essential in bacterial biofilm formation. In addition, colony detection involves the production of bacterial virulence factors, swarming, swimming and muscle contraction, and the transfer of plasmid links [92]. Signalling molecules are synthesized and secreted extracellularly by bacteria and build up in the ambient environment. As the concentration of bacteria increases, when the horizontal level of the signalling element achieves a critical threshold, the signalling elements combine with the receptor to stimulate the representation of genes associated with biofilm formation [92]. QS signalling molecules include AI-1, AI-2, and AI-3. The current study shows that the gram-negative QS system, the gram-positive QS system and the interspecific QS system are the three major QS systems. The AI-1 of gram-negative bacteria is an n-AHL. *P. aeruginosa* has two QS systems based on AHL molecules: the AHL synthase LasI synthesizes N-(3-oxodododecanoyl)-l homoserine lactone, and the AHL synthase RhlI synthesizes N-butyl-l homoserine lactone. The former is a key factor in the maturation and differentiation of biological membranes, and the latter is a key factor in the production of the biosurfactant rhamnolipid. Rhamnolipids are needed to maintain the open channel system of the *P. aeruginosa* biofilm. The PQS-based QS system releases large amounts of eDNA, and rhamnolipid synthesis is instrumental in the channelization of mushroom-like structures [93]. Earlier studies identified another signalling molecule, 2-heptyl-3-hydroxy-4-(1H)-quinolone, termed *Pseudomonas* quinolone signalling as being involved in intercellular communication in *P. aeruginosa* [94]. In their review, Jakobsen et al. summarized the current understanding of the function of colony detection in *P. aeruginosa*, stating that 0.04 to 0.06 of genes are under the control of colony detection, depending on growth conditions. These genes are responsible for the synthesis of virulence factors (protease, elastase, iron transporter, rhamnose) and are involved in colony motility and iron metabolism [49]. AI-1 of gram-positive bacteria is an autoinducible peptide (AIP), which is generally a linear or cyclic peptide composed of 5 to 87 amino acids. It exhibits amino acid modifications and lactonization that enhance AIP stability in the extracellular environment [95]. Lactic acid bacteria (LAB) first synthesize AIP precursor peptides in ribosomes, which then undergo a series of transcriptional modifications to become active AIPs. It is not capable of transporting itself through the cell wall and usually requires the atp-binding cassette (ABC) ranslocating system or other membranous pathway proteins for extracellular transport. This is said in the literature to be due to how bacteriocins are synthesized and released [96]. Inter- and intraspecific information exchange between gram-positive and gram-negative bacteria by the luxS/ai-2-mediated QS system [97]. Structurally similar ai -2 is produced by different bacteria, which enables the exchange of signals between different bacteria. In natural environments, bacteria are usually in a state of multispecies coexistence; therefore, the states of nature are usually complicated biofilms formed by more than one bacterial species [98].

Second messengers, i.e., c-di-GMP, are critical in the regulation of biofilm formation [38]. The level of intracellular c-di-GMP induces the generation of a biofilm matrix, whereas a decrease in the level of c-di-GMP leads to increased cytomotility and a transition to planktonic growth. Researchers in the latest literature have confirmed previous results that *P. aeruginosa* utilizes c-di-GMP to positively regulate the manufacture of the polysaccharides Pel and Psl, which play a crucial part in the architecture of biofilms [38, 99, 100]. *P. aeruginosa* utilizes c-di-GMP to positively regulate the manufacture of the polysaccharides Pel and Psl, which have an indispensable role in biofilm architecture. They also discovered that c-di-GMP participates in the synchronization of the adherent protein CdrA in *P. aeruginosa*. Exocytotic CdrA acts as a cross-linker of Psl chains in the substrate. In addition, cyto-associated CdrA immobilizes cells to Psl in the substratum [66]. Alginate is a polysaccharide manufactured by *P. aeruginosa*, and one of the most essential proteins engaged in the secretion of alginate production is alg44. Furthermore, the activity of alg44 requires binding of cyclic di-GMP. FleQ is a factor that activates pel transcription. PelD and FimX both regulate Pel synthesis at the posttranscriptional level and control twitching movements [93]. In contrast, another study found that the *P. aeruginosa* tyrosine phosphatase TpbA formed a link between the QS system and cellular c-di-GMP levels, which affected Pel polysaccharide production and biofilm production [101].

Sigma factors are essential regulatory molecules that participate in the general stress response. RpoS was found to be a transcription factor that positively regulates the expression of Psl [94]. In addition, sRNA is also involved in the regulation of *P. aeruginosa* biofilm formation. In this pathway, sensor kinases include GacS, RetS, and LadS; GacS can phosphorylate GacA, and GacA can activate the transcription of rsmZ and rsmY, thereby regulating the synthesis of Psl and the corresponding movement [102].

In summary, the regulatory mechanisms of adherence and biofilm formation in particular can be influenced by modified appropriate genes. Transgenic straights are regularly employed to explain the influence of diverse tools (antibiotics, chemicals disrupting signalling elements, etc.) on the process of biofilm formation [49].

## Mechanisms underlying biofilm dispersion

In accordance with the mechanisms of biofilm formation, bacteria have additional mechanisms for dissipation from biofilm dispersion [80]. These processes include reduction of microbial adhesion, decomposition or modulation of the biofilm substrate (Fig. 3B). Cell migration in biofilm colonization is an essential condition for the generation of new colonies in new locations and can be triggered if biofilm cells are exposed to negative elements [49].

Localized diffusion of *P. aeruginosa* biofilms in LB medium-fed flow chambers enables the observation of some hollowed out microcolonies [49]. At the initial stage of the diffusion process, cell wall subpopulations formed by nonmotile cells form the exterior of the microcolony, and fast-moving cell wall subpopulations are present in the interior of the microcolony. The motile subpopulations eventually find their way out of the microcolony, thus resulting in a small population with a central cavity. This dispersion phenomenon is determined by whether the microcolony reaches a threshold size or not [103].

The dispersion response of *P. aeruginosa* biofilms to changes in carbon supply was reported by Sauer et al. When there is a sudden increase in carbon supply, Microcystis aeruginosa biofilms grown in glutamate flow chambers initiate a dispersion process that results in the release of a large portion of the biomass from the biofilm [66]. The degree of dispersion depends on the carbon source and is associated with increased flagellar expression and impaired contractile movement. A consequent study identified gene products in *P. aeruginosa* involved in the detection of ecological factors that trigger biofilm dispersion [104]. Sequence analysis and phenotypic comparison of wild-type and bdlA mutants of Microcystis aeruginosa revealed that the BdlA protein is a chemotactic regulator that affects intracellular c-di-GMP levels. There is increasing evidence that the production of stromal components, such as Pel/Psl polysaccharides and vesicular cilia, is regulated by proteins containing either double-positive (guanylate cyclase) or phosphodiesterase enzymes that control intracellular c-di-GMP levels [38].

In contrast, a high intracellular concentration of c-di-GMP upregulates substrate formation and periplasmic membrane formation, whereas a low intracellular concentration of c-di-GMP downregulates substrate formation and induces zooplanktonic lifestyles [70]. Carbon starvation and nitric oxide signalling induce diffusion through *P. aeruginosa* biofilms by inducing phosphodiesterase activity, leading to a decrease in intracellular c-di-GMP levels. There is evidence indicating that the abovementioned BdlA chemoregulators are involved in nitric oxide-mediated biofilm diffusion. Rhamnolipids play several roles in the developmental cycle of the *P. aeruginosa* biofilm, including the production of large amounts of *P. aeruginosa* that may lead to cellular dispersion from the biofilm [105]. Recent studies have shown that rhamnolipid-mediated diffusion in *Pseudomonas aeruginosa* biofilms may be involved in c-di-GMP. It is evident that the PA2572 protein degrades inactivated c-di-GMP phosphatidylcholine (phosphatidylcholine), which is a key component of biofilm development. There is evidence that the PA2572 protein has a structural domain of degradation-inactivated c-di-GMP phosphodiesterase, which may have a regulating role [81, 103].

In addition, it has been demonstrated that cis-2-decenoic acid, a compound produced by *P. aeruginosa*, induces the diffusion of formed biofilms and inhibits biofilm formation [81]. Exogenous addition of cis-2-decenoic acid at natural concentrations to *P. aeruginosa* biofilms induced biofilm microcolony diffusion. The molecule also induced the diffusion of biofilms formed by *E. coli, Klebsiella pneumoniae, Proteus mirabilis, Streptococcus pyogenes, Bacillus subtilis, S. aureus* and *Candida albicans* [38, 106]. The authors concluded that Microcystis aeruginosa produces cis-2-decenoic acid continuously as it grows in a biofilm and that small colonies do not diffuse because cis-2-decenoic acid is removed by diffusion and advective transport; however, larger microcolonies diffuse because cis-2-decenoic acid production exceeds the rate of diffusion [99].

In *P. aeruginosa*, the large outer membrane adhesion protein LapA mediates the attachment of surface and matrix components [107]. The release of LapA from the cell surface leads to biofilm spreading, which is mediated by the activity of the extraplastidial protease LapG [100]. LapG protease activity is regulated by the transmembrane protein LapD, harboring a c-di-GMP-binding construct; LapD contains a c-di-GMP-binding construct that inhibits LapG at high levels of c-di-GMP, whereas LapD inhibits LapG at reduced concentrations of c-di-GMP [78]. Current data indicate that a comparable system operates in *P. fluorescens*. *P. aeruginosa* codes for a number of bulky adhesion proteins but has no lapA isoforms [76]. Nevertheless, *P. aeruginosa* indeed has congeners of lapD and lapG; therefore, mechanisms similar to those of *P. putida* may be involved in *P. aeruginosa* biofilm diffusion.

## Biofilms and diseases

The formation of biofilms confers a protective shield upon microorganisms, defending them against host immune system assaults and facilitating their colonization and proliferation, thereby ultimately fostering the establishment of chronic infections. This protective mechanism not only limits the impact of antibiotics but also heightens the challenge of treating the infection. The presence of biofilms is closely linked to the onset of diseases across various organs throughout the body [108].

### Respiratory/pulmonary infection

Biofilms are associated with many chronic pathogenic bacterial infections in the respiratory system, such as bronchiectasis, chronic obstructive pulmonary disease (COPD) and cystic fibrosis [109]. Cystic fibrosis is a pulmonary ailment triggered by lung infections that are primarily induced by bacteria such as *P. aeruginosa*, *S. aureus*, *Haemophilus influenzae*, or *Burkholderia cepacia*. The formation of biofilms enables these microbial complexes to resist host defenses and withstand antibiotic treatments, thus leading to prolonged survival within the patient's respiratory tract [110]. Persistent pulmonary infections linked to *Pseudomonas* and *Mycobacterium* are also correlated with COPD as well as bronchiectasis not induced by cystic fibrosis [111, 112]. The causative agent of tuberculosis is *M. tuberculosis*. When *M. tuberculosis* forms a biofilm, the cords within alveolar cells play a role in suppressing innate immune signalling through nuclear compression [113]. Beyond chronic conditions, biofilm-induced infections can also precipitate acute respiratory ailments, including ventilator-associated tracheobronchitis (VAT) and ventilator-associated pneumonia (VAP) [114].

### Oral

The oral cavity, with its unique blend of humidity, temperature, and nutrient-rich conditions, is home to a rich and diverse microbiome and is one of the five major bacterial reservoirs in the human body. When the oral microbiome is imbalanced, special flora become the dominant bacteria. These microorganisms aggregate to form oral biofilms upon colonization of tooth surfaces or restorations. This is the precursor to various oral maladies, including dental caries, periodontitis, wisdom tooth pericoronitis, jaw osteomyelitis, and pulp infections [115]. When the relationship between biofilms and the host is severely imbalanced, systemic diseases may occur, such as diabetes, cardiovascular and cerebrovascular diseases, kidney disease, and respiratory disease [116]. Previous research has indicated that targeting subgingival biofilms is an effective strategy for treating periodontal diseases. Aseptic animal experiments underscore the role of microbial interactions in inducing destructive periodontal bone loss [117]. Moreover, oral flora have been found to activate a group of immune cells that in turn influence osteoclasts. Further animal experiments found that using antiseptic mouthwash to consume healthy oral flora can effectively alleviate naturally occurring bone loss and maintain alveolar bone homeostasis [118]. The subgingival bacteria associated with periodontitis play a regulatory role in the spatial structure and pathogenicity of the supragingival biofilm. These pathogens often interact with each other, leading to microbial dysbiosis and enhanced interplay with the host's inflammatory response, collectively contributing to the onset of the disease [119]. The corncob structure of dental plaque (consisting of spherical bacteria tethered to coryneform filaments) can be used to analyze the microhabitat community structure in complex natural biofilms. Using fluorescence in situ hybridization and spectral imaging, researchers found that within the corncob structure of dental plaque, each taxonomic group is associated with a limited potential microbiota, which could promote the long-term stability of the population [120]. In-depth elucidation of neighboring bacterial relationships in oral biofilms can enable targeted regulation of communities to maintain oral health as well as systemic health.

### Urinary system

Urinary tract infections rank among the most prevalent infections, and they can be caused by both gram-positive and gram-negative bacteria. Gram-negative urinary tract pathogens include *E. coli*, *Proteus mirabilis*, and *P. aeruginosa*, among others, with *E. coli* being the most common pathogen [121]. These pathogens possess surface structures that recognize specific host cells. Upon firmly attaching to catheters, these bacteria undergo transformative changes, culminating in the formation of biofilms that shield the pathogen from both antibiotics and the host's immune response [122]. Researchers have made notable strides in combating biofilms associated with urinary tract infections. Specifically, studies suggest that the combination of aminoglycosides with specific metabolites holds promise in treating biofilms formed by *E. coli* and *S. aureus*. This innovative approach is effective at enhancing the treatment of chronic infections, as evidenced by positive outcomes in mouse models of urinary tract infection [123]. A novel drug delivery system has recently been developed by employing protein-based nanofilms assembled on catheters. This system ensures stable and controllable drug delivery while exhibiting outstanding antibiofilm properties [124].

### Gastrointestinal tract

Beyond the contexts previously discussed, biofilms also play a significant role in gastrointestinal diseases. Bacteria forming biofilms in the gastrointestinal tract (GI) have advantages in terms of both quantity and metabolism. They envelop themselves in the formed EPS matrix for protection, and they participate in chronic GI infections [125]. Biofilm formation and long-term colonization are major contributors to foodborne diseases [126]. These biofilms can manifest along the entire length of the gastrointestinal tract, contributing to a spectrum of conditions. The development of biofilms is closely linked to diseases such as colorectal cancer, inflammatory bowel disease, and gastric infections, thus underscoring the broad impact of biofilm formation within the gastrointestinal system [127]. *Helicobacter pylori* is the most common cause of duodenal ulcers, gastric ulcers, and gastric cancer. The onset of these diseases appears to be strongly linked to the colonization of the stomach by *H. pylori*-induced biofilms [128].

### Wound

Biofilms are strongly associated with wound conditions. They play a significant role in both chronic wounds and, to a lesser extent, acute wounds. In particular, chronic wounds exhibit an enduring association with biofilms. In the case of chronic wounds, biofilms continuously interact with components of the host immune system and significantly extend the typical inflammatory phase of wound healing, thereby disrupting the natural course of the healing process [129]. This prolonged interaction contributes to the inherent challenges in the self-healing capacity of chronic wounds. Simultaneously, the EPS mechanism of biofilm formation serves as a barrier against the penetration of antibiotics and other therapeutic drugs. This protective function enhances the development of internal bacteria and poses challenges to antibiotic-based treatments [130]. Addressing open wounds requires a multifaceted approach that involves antimicrobial therapy coupled with debridement. This combined strategy serves to effectively control wound infection and manage the impact of biofilms, thereby promoting a more conducive environment for the wound healing process [131].

## Antimicrobial therapeutic strategies based on biofilms

**Fig. 4:**
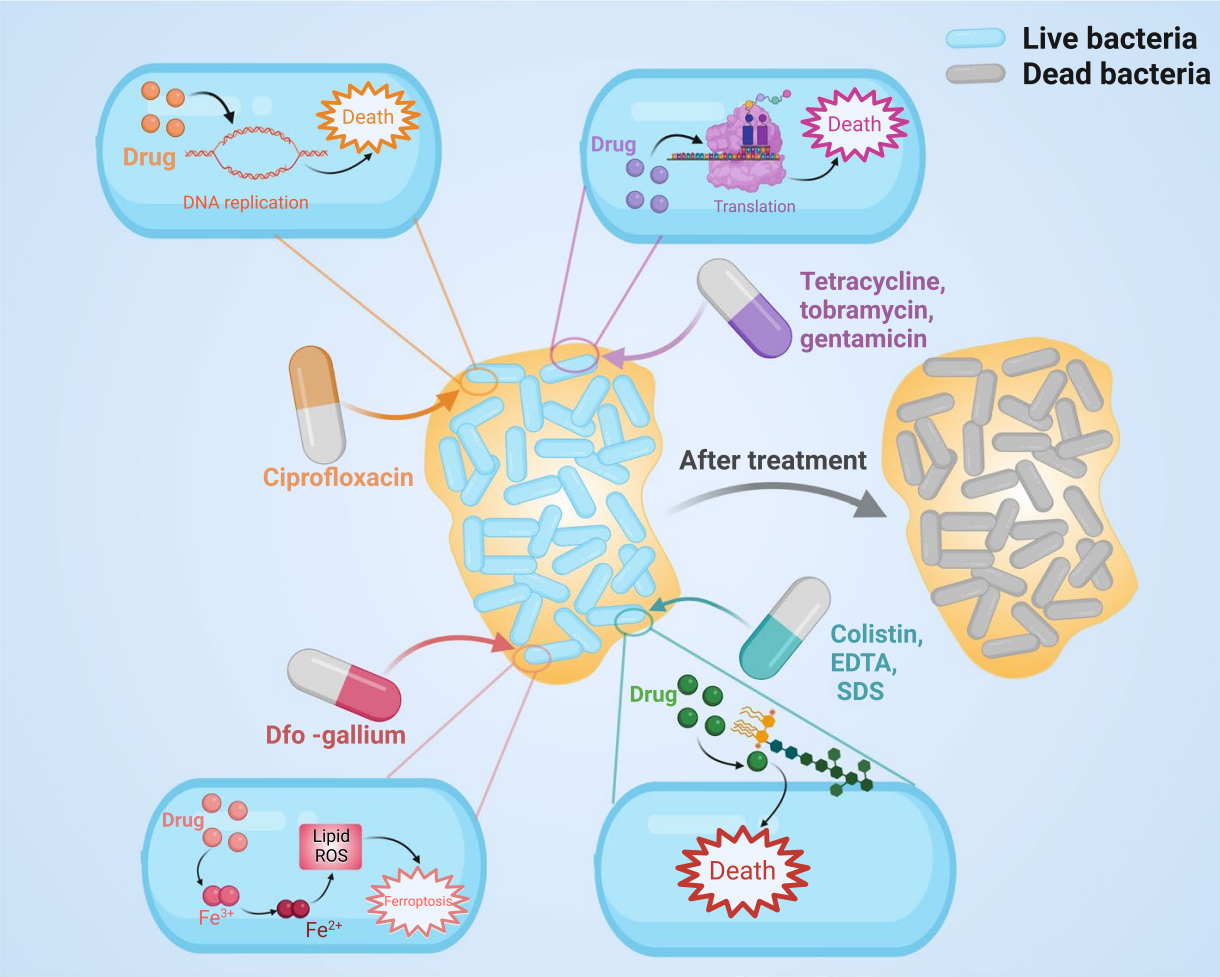
Mechanisms of drug action. Different antibiofilm drugs have different mechanisms of action. For example, ciprofloxacin works by interfering with bacterial DNA replication, while tetracycline, tobramycin and gentamicin interfere with translation, all of which specifically kill cells that are metabolically active at the top of the biofilm. Dfo-gallium is antibacterial by interfering with the iron metabolism of cells, while colistin, EDTA and SDS destroy the structure of the membrane by combining with the lipopolysaccharide terminal lipid outside the bacterial membrane, and these two kinds of drugs kill the cells in the deep biofilm

Microbial biofilms are able to develop tolerance when the host immune system or antibiotics act on bacteria. This ability is one of the most important characteristics of biofilms [126, 132]. Since antimicrobial drugs can only reduce the number of bacteria but not eradicate them, bacterial infections still have the possibility of infection recurrence after antibiotic treatment [133]. Therefore, we have an urgent need to develop targeted drugs against biofilms in the clinic. In this section, we summarize the information about antibiofilm drugs (Table 2) and propose potential antimicrobial therapy strategies based on current knowledge. For example, ciprofloxacin works by interfering with bacterial DNA replication, while tetracycline, tobramycin and gentamicin interfere with translation, all of which specifically kill cells that are metabolically active at the top of the biofilm. Dfo-gallium exhibits antibacterial activity by interfering with the iron metabolism of cells, while colistin, EDTA and SDS destroy the structure of the membrane by combining with the lipopolysaccharide terminal lipid outside the bacterial membrane; these two kinds of drugs kill the cells in the deep biofilm [134] (Fig. 4).

**Table 2:** Information about antibiofilm drugs

**Class**	**Representative drug**	**Advantages**	**Disadvantages**	**Ref.**
Conventional β-lactams	Piperacillin-tazobactam, ceftazidime, imipenem	Strong antibacterial activity against *P. aeruginosa*	Drug resistance	[85]
Fluoroquinolones	Ciprofloxacin, levofloxacin and Prilosec	The only class of anti-*Pseudomonas* drugs that can be used as oral formulations	Cause resistance through the efflux pump; the antibacterial activity will be inhibited in the acidic medium.	[135]
	Difloxacin and delafloxacin	The antibacterial activity will be enhanced in acidic medium.	Possible hypersensitivity reactions	[136]
	Finafloxacin	The use of ear suspension in the therapy of acute otitis externa caused by *P. aeruginosa*	There may be reactions such as diarrhea, nausea, headache and nasopharyngitis	[137, 138]
Aminoglycoside	Tobramycin	An inhalant that is effective in treating and preventing infections in individuals with chronic lung disease	It should be avoided as a single drug unless it is used to treat urinary tract infections	[139]
	Plazomicin	Effective against a majority of bacteria containing glycoside-modifying enzymes in aminoglycosides	Inability to resist resistance mechanisms associated with the expression of membrane permeability or exocytotic pumps and acts only on urinary tract infections	[140, 141]
Polymyxin	Visfatin, fucoxanthin	It can be injected intravenously or inhaled by nebulization; it has higher antibacterial effect than intravenous administration	Limited by nephrotoxicity and neurotoxicity.	[142, 143]
New β-lactams	Ceftolozane/tazobactam	Optimal antimicrobial activity is not affected by outflow of activity or pore changes; can be used for empiric or targeted treatment of MDR-PA	Narrow-spectrum	[144, 145]
	Cefiderocol	A novel siderophore cephalosporin; shows the strongest in vitro activity against IRPA	More adverse reactions	[146]
	Fosfomycin	It can be taken orally to treat urinary tract infection	Bacteria will quickly develop drug resistance after contact, and it is recommended to be used in combination with other drugs	[147, 148]
	Murepavadin	The first member of the outer membrane protein that targets antibiotics and can resist some mechanisms of drug resistance	Not widely used	[149]
Bacteriophages	Bacteriophages	Harmless to humans and entirely unique to the host bacteria	Limited spectra	[150, 151]
Nanoparticles	Antibiofilm Nanoparticles	Providing promising therapeutic delivery platforms and effective biofilm targeted approaches	Need to improve in vivo efficacy and biocompatibility	[152]

### Conventional antibiofilm drugs

#### Conventional β-lactams

Penicillin with b-lactamase inhibitors is extremely effective in the treatment of *P. aeruginosa*, which is the mainstay of conventional β-lactams. Ceftazidime, cefoperazone and cefepime are among the cephalosporins that are resistant to *P. aeruginosa*. Among the carbapenems, doripenem is the most effective against *P. aeruginosa,* which has the lowest minimum inhibitory concentration (MIC) value in its class of drugs [153].

While conventional β-lactams are the mainstay of empirical treatment of *P. aeruginosa*, local etiology ought to be one of the most critical decisive factors that guide the choice of therapy. The China Antimicrobial Resistance Surveillance System (CARSS) showed that in China, the average prevalence of *P. aeruginosa* resistance to carbapenems was 18.3% in 2020, while the detection rate of carbapenem-resistant *P. aeruginosa* (CR-PAE) was 18.3% in 2020. A slowly decreasing trend was observed in the last three years of the study. Bacterial resistance may develop during therapeutic treatment with β-lactams [154]. In a cohort study of 271 patients treated for *P. aeruginosa* infections, ceftazidime had the lowest risk of developing resistance during treatment, while imipenem had the highest risk of developing resistance. Imipenem can be used to treat *P. aeruginosa* infections based on in vitro drug sensitivity testing. Imipenem is an attractive option for patients who are allergic to penicillin or have developed resistance to other drugs. It has been studied in patients with chronic lung disease and cystic fibrosis. A randomized controlled trial (RCT) including 211 patients with cystic fibrosis showed a delay in the need for inhaled or intravenous antipseudomonas antibiotics and improvements in respiratory symptoms and lung function after 28 days of imipenem inhalation [155].

#### Fluoroquinolones

Ciprofloxacin, levofloxacin and prilosec are quinolones that have been shown to reliably exhibit activity against *P. aeruginosa*. They are represented in the only group of anti-*Pseudomonas* drugs that can be used as oral preparations. Nevertheless, resistance may develop with the utilization of these drugs, mainly through efflux pumps [135]. Difloxacin and delafloxacin are two novel fluoroquinolones that show enhanced antipseudomonal activity in *acid* media [136]. It is an essential signature, as a low pH inhibits the antimicrobial activity of other fluoroquinolones, thus enabling research on infections caused by novel drugs at acidic sites, such as UTI and gastritis [156]. In 2014, U.S. The Food and Drug Administration authorized fenafloxacin to be made into an ear suspension for the treatment of acute exophthalmos due to *P. aeruginosa* [137]. A phase II RCT in complex UTI comparing the efficacy of fenofloxacin treatment for 5, 10 and 10 days showed that fenofloxacin treatment for 5 days was superior in terms of virtual clinical amelioration and microbial eradication. Disulfiram was authorized in 2017 for the therapy of skin and soft-tissue infections [139, 140]. Evidence suggests that both drugs hold great promise for the future treatment of *P. aeruginosa.*

#### Aminoglycosides

Tobramycin, amikacin and gentamicin are efficacious against *P. aeruginosa*. However, they are to be avoided as single agents, except for the therapy of UTIs [139]. Inhaled agents, particularly tobramycin, have been demonstrated to effectively treat and prevent infections in individuals with chronic lung diseases such as bronchiectasis and cystic fibrosis. Studies of inhaled tobramycin for the treatment of noncystic fibrosis brucellosis are currently underway. Plazomicin is a novel aminoglycoside that is active on the majority of bacteria containing aminoglycoside-modifying enzymes, including carbapenem-resistant strains. Although its MIC activity is below that of other aminoglycosides, it is lower for infections due to *Pseudomonas* than for infections due to *Enterobacteriaceae* [140]. Nevertheless, this drug does not prevent resistance mechanisms related to membrane permeability or the expression of efflux pumps, so its action has thus far been limited to UTI therapy [141].

#### Polymyxins

Mucins and polymyxin B as potential agents for the management of multidrug-resistant *P. aeruginosa* (MDR-PA) when other therapy options are restricted. A comprehensive systemic review demonstrated that intravenous and/or nebulized inhalation of fumonisins is a safe and effective therapeutic option for the treatment of VAP associated with gram-negative bacteria, including *P. aeruginosa* [142]. Other research on intravenous fucoidan in critical patients with VAP due to drug-resistant gram-negative bacilli has shown that intravenous fucoidan fails to penetrate the intraepithelial fluid (ELF), leading to substandard concentrations of fucoidan in the alveolar fluids of the bronchial tubes [157]. However, aerosols produce appropriate concentrations of ELF with higher antimicrobial potency than intravenous administration [143]. The administration of these drugs is restricted by renal toxicity and neurotoxicity.

#### New β-lactams

Cefazolin/tazobactam (C/T) is a compound of a unique novel cephalosporin and an elderly β-lactamase inventor. The activity of cefazolin is superior to that of most beta-lactam drugs against *P. aeruginosa* because it is stable toward the AmpC enzyme produced by *P. aeruginosa* and is independent of active ectopic flow or pore changes [144]. This drug can be utilized as an empiric or targeted therapeutic for MDR-PA [158]. It has been authorized for the management of UTIs, endoabdominal infections, and hospital-acquired pneumonia [159]. In an in vivo assay of 42 CR-PA separates, C/T was efficient for almost all separates [145]. A multicenter retrospective study demonstrated that initiating C/T for MDR-PA within 4 days of culture acquisition was related to an improvement in overall survival, clinical success, and microbiological cure rates [159]. In a separate retroactive trial of 21 patients, C/T succeeded in treating 71% of individuals with MDR-PA, but 3 individuals became resistant to the drug [145]. C/T had reduced activity against carbapenemase-producing CR-PA strains [160].

Cefiderocol is a novel glycophilic cephalosporin with anti-MDR-PA activity. In a clinical phase II trial studying the management of MDR urinary tract pathogens, including *Pseudomonas* spp., cefiderocol was shown to be nonsuperior to imipenem in efficacy [161]. In a previous study, it exhibited the greatest in vitro activity toward imipenem-resistant *P. aeruginosa* (IRPA) in comparison with C/T and C/A [146].

Fosfomycin is an ancient antibiotic that is typically administered orally for the treatment of urinary tract infections. In many countries, fosfomycin is administered intravenously and shows potent activity against *P. aeruginosa*, including MDR-PA [147]. Since *P. aeruginosa* rapidly acquires resistance after contact with fosfomycin, it is only proposed as part of a combination therapy. An in vitro investigation of 15 MDR-PA isolates revealed a positive synergistic effect of fosfomycin with carbapenems in more than half of the cases [148, 162].

Murepavadin is a novel external membrane protein-targeted antibiotic [149]. This property enables the drug to target this resistance regime in *P. aeruginosa* and is very effective toward CR-PA, C/T resistance and mucin resistance.

#### Bacteriophages

As natural enemies of bacteria, phages can be used as an alternative to antibiotics or antibiotic supplements to treat infections. Phages are harmless to humans and completely unique to host bacteria. Studies have shown that biocontrol of bacteria by phages, phage-encoded enzymes (EPS digestion), and purified phage-encoded enzymes (bacterial cell wall digestion) reduces biofilm formation and decreases bacterial density in animal tissues [58, 150]. For example, T4 phages can colonize biofilms formed in *E. coli* and disrupt the physical structure of the biofilm by killing the bacteria [163, 164]. A number of bacteriophages generate polymerases that can degrade the intracellular matrix. It has also been found that Klebsiella can use capsules to degrade phages to produce gas and that *E. coli* O157 and *Salmonella enterica* can use three different phages in chickens [151]. However, the host specificity of phages also limits their efficacy*,* as they have a limited spectrum. In addition, there have been findings of phage resistance in bacteria. The other drawback of phage therapy is that some phages bear genes for invasion factors that are transmitted to *bacteria* treated by phages [163]. Nevertheless, phospho therapies are currently employed as among the adequate methods to inhibit drug-resistant bacteria. It is assumed that phage therapeutics hold tremendous future value in the therapy of bacterial infection *due to* the significance of drug tolerance, *particularly* to the strains of organisms that produce biofilms [165].

#### Nanoparticles

Nanotechnology is a technology that involves the creation of new materials, tools and systems by controlling them at the level of molecules and atoms. Recently, nanotechnology has emerged as a promising instrument for the prophylaxis and manipulation of biofilms [150]. By coating surfaces with nanoparticles, biofilm formation can be prevented [152]. This may be attributed to the repression of bacterial adhesion to the surface, to the antimicrobial properties of the nanoparticles, or a combination of both [152]. Nanomagnesium fluoride has antimicrobial characteristics and prevents biofilm formation of important pathogens such as *E. coli* and *S. aureus*. Catheters coated with this material are strongly susceptible to biofilm infections associated with these pathogens [150]. In addition, nanosilver has been shown to enhance the causative properties of ampicillin and vancomycin toward gram-negative and gram-positive bacteria [150]. More recent findings have confirmed the strong antimicrobial and *antibiofilm* potential of nanoparticles for use in food packaging materials, polymeric matrices on surfaces, and especially medical devices [152, 166].

### Emerging antibiofilm agents

The ability of biofilms to resist unfavorable ecological circumstances and overcome the present host's immune system has prompted the search for new antibiofilm drugs. Novel antibiofilm drugs, such as micromolecular inhibitors, legal quorum inhibitors, microbial peptides, efflux pump inhibitors (EPIs), quaternary ammonium compounds (QACs), and native botanicals, selectively act through different mechanisms and fight drug resistance. These drugs can target extraterminal substrates for formation, facilitate biofilm dispersion or act on elastic cells at the core of the biofilm. Methods to improve the curative effect of antibiofilm agents involve encapsulating the drug in nanoparticles to achieve the best effect of delivery or mixing several agents to enhance the antimicrobial activity. Nevertheless, the internal cytotoxicity and therapeutic effect of antibiofilm drugs remain a major concern. Table 3 lists the current major or emerging antibiofilm agents, and these agents are further discussed in the subsequent sections.

**Table 3:** Summary of antibiofilm agents identified against biofilm-associated resistant infections

**Antibiofilm drugs**	**Target pathogenic bacteria**	**Antibiofilm mechanism of action**	**Research Model**	**Reference**
**Exopolysaccharide-targeting drugs**				
Quinoxaline derivative	*Streptococcus mutans*	Glucosyltransferase inhibitor	Anticaries rat	[167]
Oxazole derivative	*S. mutans*	Antagonizing glucosyltransferases	Dental caries rat	[168]
Dispersin B	*Staphylococcus* spp.	Inhibiting skin colonization by inducing staphylococci to detach from the skin	In vivo pig model	[169]
Endolysins	*S. aureus*	Peptidoglycan hydrolases	System MRSA infection in mice	[170]
Dornase alfa	*Pseudomonas aeruginosa*	Dissolution of cystic fibrosis sputum and fibrous structures	Cystic fibrosis sputum	[171]
DNABII antibodies	*Haemophilus influenzae*	DNABII epitope targeted to external cellular DNA	Chinchilla and murine	[172]
α-amylase	*S. aureus* and *P. aeruginosa*	Destruction of exopolysaccharides	*Danio rerio*	[173]
**Biofilm dispersion-targeting drugs**				
Nitric oxide	*P. aeruginosa*	Biofilm dispersal and reduction of biofilm antibiotic tolerance	Cystic fibrosis sputum	[174]
Cephalosporin-3′-diazeniumdiolates	*P. aeruginosa*	Biofilm dispersion; enhance biofilm sensitivity to antibiotics	Microtiter plates	[175]
Nitroxides	*P. aeruginosa*	Promotes biofilm dispersion, inhibits biofilm formation, increases swarming motility	Flow chambers	[176]
Autoinducing peptide inhibitor	*S. aureus*	Quorum sensing inhibitor	RN9222 cell line	[177]
Natural peptide Capsicumicine	*S. epidermidis*	Decomposing biofilm substrates	SKH1 mice	[178]
**Biofilm persister-targeting drugs**				
TM5 peptide	*P. aeruginosa* and* S. aureus*	Anti-inflammatory antiseptic	Laboratory settings	[179]
Rifampin + Fosfomycin	*S.aureus* (Methicillin-resistant)	Curing cage related infections	A foreign body infection model using guinea pigs	[180]
Acyldepsipeptide ADEP4	*S. aureus*	Activates ClpP protease to kill growing and stubborn cells	Mouse model of a chronic infection	[181]
Glycosylated cationic peptides	*S.aureus* (Methicillin-resistant)	Sterilization of recalcitrant cells and dispersion of biofilm masses	Ex vivo wounded human skin infection	[164]

#### Targeting extracellular polymeric substances

*Small Molecule Inhibitors* Small molecule inhibitors associated with biofilms include inhibitors that target the synthesis of intracellular signaling molecules (second messengers) commonly found in bacteria, such as c-di-GMP, which modulates EPS-producing enzymes in gram-positive (e.g., *Corynebacterium* mutans) and gram-negative (e.g., *P. aeruginosa*) bacteria. Therefore, small molecule inhibitors can be used to block c-di-GMP production as a strategic approach to combating biofilms and related infections. In in vitro biofilm models, small molecule inhibitors of diguanylate or diadenylate cyclase (e.g., catechol-containing sulfonylhydrazine compounds) have been identified as potent biofilm antibiotics, but their efficacy as antibiofilm agents in vivo needs further validation [182]. An alternative method is to use an inhibitor of glycosyltransferases (e.g., quinoxaline derivatives) to inhibit the synthesis of dextran EPS by glycosyltransferases, thereby reducing the concentration of pathogen biofilms on teeth and preventing dental disease [167]. The drug that disrupts the biofilm formed by this species of fungus is an insecticide that is designed to inhibit the association of Candida with host fibronectin [167]. Biofilm cells can attach to EPS within mature biofilms, and a variety of biological molecules that combine with EPS bonding factors are being investigated as possible therapies for biofilm infections. They provide prospective routes for the development of novel treatments targeting bacterial biofilms related to sustained infections.

##### Enzymes degrading extracellular polymeric substances

Depending on the chemical structure of exopolysaccharides, *the* degradation of exopolysaccharides can be an approach of great significance in the fight against bacterial biofilms. Methods range from the destruction of the substrate of pathogenic oral biofilms using exopolysaccharide-degrading enzymes (e.g., Disensin B) to the use of dextran hydrolases for the degradation of EPS related to oral biofilms in dentistry [182]. An alternative method is to use the privatized serine enzyme Esp to suppress *S. aureus* biofilm formation and eliminate original biofilms in vitro to increase the susceptibility of the cells forming the biofilm to the antibacterial agent β-defensin 2 and to reduce *S. aureus* colonization of the human nasal cavity [167].

#### Antibody and nucleic acid-binding proteins

EPS-targeted antibodies and nucleic acid-binding proteins can be available for biofilm infections. Despite the fact that the administration of vaccines presents quite a challenge in terms of their utilization based on the antigenic vagrancy of clinical separates of biofilm-forming strains, the employment of polyclonal antibodies directed against particular EPS constituents has demonstrated hope. *P. aeruginosa*-specific antibodies increase opportunistic killing of *P. aeruginosa* and inhibit pathogen adhesion to pulmonary epithelial cells; furthermore, in some animal models, such antibodies offer preventive protection *against P. aeruginosa* infection [182]. Immunotherapy aimed at the DNA-binding protein DNABII, in combination with antibiotic treatment, was effective in vivo in mammalian lung infection models targeting biofilms of a wide range of bacteria [172]. *Furthermore,* joint immunization and antibiotic treatment proved to be effective on methicillin-resistant *S. aureus* (MRSA) biofilms [183]. In addition, the combination of DNABII antibodies with a comprehensive host factor-targeted vaccine disrupts *nondifferentiated Haemophilus influenzae* biofilms and prevents relevant diseases [184]. The combined immunization method targeting several targets *has potential* for the prevention of biofilm-associated infections [182].

#### c-di-GMP biosynthetic inhibitors

C-di-GMP *plays* a critical *role* in the biofilm life cycle of the vast majority of bacteria, and therefore therapeutic approaches aiming at the metabolic pathway of c-di-GMP constitute a viable therapeutic strategy, *although* the intricacies of the regulation of c-di-GMP render it a challenge to manage [182]. *Nitric* oxide modulates the level of c-di-GMP concentration and facilitates the dispersion of biofilms [174, 185]. Cephalosporin-3'-diazodimers (C3Ds) are prospective agents for biofilm dispersion that preferentially transport NO to bacterial biofilms*,* as bacterial β-lactamases cleave the β-lactam ring and liberate NO. C3Ds have been demonstrated to be efficient at disrupting *P. aeruginosa* biofilms [175]. Due to the instability of NO, nitroxide analogs are currently in the process of being developed as NO substitutes and to ensure that they can fulfill their *antibiofilm* potential [176, 186]. In summary, current research suggests that the mechanism of action of c-di-GMP biosynthesis inhibitors involves facilitating the disintegration of biofilms, allowing bacteria to be more readily eliminated by conventional antibiotics, thereby reducing the likelihood of bacterial recolonization and decreasing the probability of recurrent infections [187, 188].

#### Quorum sensing inhibiting peptides

Targeting group sensing, a strategy that involves interfering with bacterial intercellular communication systems, is a *prospective* method for the discovery of innovative antibiotic membrane therapeutics [189, 190]. The role of group-sensing inhibitors (QSIs) in clinically relevant bacterial biofilms has been extensively evaluated by in vitro and in vivo models. For instance, a self-inducing peptide inhibitor was developed to effectively reduce subcutaneous biofilm formation in a murine model of transplantation [177]. Recent studies have shown that the mechanism of action of antimicrobial peptides involves disrupting the bacterial QS system and repressing the production of virulence factors, biofilm formation, and EPS accumulation to treat bacterial infections [183]. In addition, the human hormone atrial natriuretic peptide can strongly disrupt *P. aeruginosa* biofilms by targeting AmiC*-*sensing proteins by direct action on *P. aeruginosa*, and the peptide potentiates the *antibiofilm* properties of a wide range of antibiotics [184].

## Conclusions and perspectives

Biofilms are complex microbial communities that inhabit polysaccharide and/or protein matrices that can be generated by a wide range of microorganisms, comprising a variety of bacteria and fungi [191]. The antimicrobial resistance of cells in biofilm constituents is caused by the structural properties of biofilms and phenotypic alterations of sequestered cells and is now a major challenge in clinical therapy [192]. Bacterial biofilms have an impact on all dimensions of human health, primarily in the form of bacterial infections, ranging from long-term infections, dental plaque, and infections resulting from retention of medical appliances. They can likewise cause considerable problems for other industries, such as oil extraction, water storage, paper, metal processing and food manufacturing [193]. Biofilms are a major contributor to chronic infections. Typically, bacteria that tend to form biofilms are mostly members of the genus *Pseudomonas* [194]. In the last few years, humans have gained a great deal of practical knowledge in the treatment of bacterial biofilms. It should be noted, however, that most of the treatments are not generalizable, and the appropriate therapy depends on the site of biofilm formation [187–189].

In medical devices, the development of new materials with antiadhesive properties may be most valuable to prevent biofilm formation. In addition, if it is possible to find a certain natural compound and understand the principle of their regulatory mechanism that interferes with biofilm formation, and at the same time it does not promote the birth of superresistant bacteria, we can make a major breakthrough in this field and contribute to clinical biofilm-targeted therapies [132, 195]. Since the process of biofilm formation often involves complex mechanisms that are independent of each other, this is an extremely challenging task, and it is hoped that future breakthroughs in this area will be made by aspirants of interest [196, 197]. At present, it seems that the mechanism of cyclic di-GMP action during biofilm formation is incompletely understood. A theoretically feasible approach would be to combine nanomaterials with natural active substances, as both have antibiofilm activity, and we hope that the combination of the two would be competent for the clinical treatment of biofilms [191, 198].

Bacteria in biofilms are more viable than free-floating bacteria because they can utilize surface adhesion and biofilm formation to increase their survival rate [199, 200]. A number of defense strategies seem to be inherent in biofilm formation, such as inhibition of substrate release, reduced metabolism due to nutrient limitation, development of dormant remnants, and an increase in oxidative stress; moreover, it appears that all of these phenomena can affect the building up of protective settings [201, 202]. Various strategies emerge as a result of factors both inside and outside the biofilm, involving antibiotic-induced gene exposure in its cells. Nevertheless, there must also be other strategies for adding antibiotic resistance [203, 204]. What is clear is that more research is needed to reveal the rationale for targeting drugs against biofilms, as the multifactorial aspects of bacterial resistance in biofilms have hindered our identification of these pathways. It is hoped that future discoveries in these areas will bring better novel therapies for biofilm-associated infections [205–207].

## Data Availability

Not applicable.

## Abbreviations

pH: Potential of hydrogen
Lap: Leucine aminopeptidase
c-di-GMP: Cyclic dimeric guanosine monophosphate
Psl: Polysaccharide synthesis locus
Pel: Pellicle polysaccharide
eDNA: Extracellular DNA
CF: Cystic fibrosis
EPS: Extracellular polymeric substance
RSCV: Rugose small colony variant
PQS: *Pseudomonas* quinolone signal
LPS: Lipopolysaccharide
MV: Membrane vesicles
QS: Quorum sensing
TCS: Two-component signal
HK: Histidine kinase
RR: Response regulator
RT‒qPCR: Real time quantitative
AHL: Acyl homoserine lactone
AIP: Autoinducible peptide
LAB: Lactic acid bacteria
ABC: Atp binding cassette
sRNA: Small RNA
EDTA: Ethylene diamine tetraacetie acid
SDS: Sodium dodecyl sulfate
MIC: Minimum inhibitory concentration
SMART: Study to monitor antimicrobial resistance trends
IUHIC: International union for hospital infection control
CR-PA: Carbapenem-resistant *P. aeruginosa*
CARSS: China antimicrobial resistance surveillance system
CR-PAE: Carbapenem-resistant *Pseudomonas aeruginosa*
RCT: Randomized controlled trial
UTI: Urinary tract infections
FDA: Food and Drug Administration
MDR-PA: Multiple-Drug Resistant* Pseudomonas aeruginosa*
VAP: Ventilator-associated pneumonia
ELF: Intraepithelial fluid
C/T: Ceftolozane/tazobactam
C/A: Ceftazidime/avibactam
EPI: Efflux pump inhibitor
QAC: Quaternary ammonium compound
MRSA: Methicillin-resistant S.aureus
C3D: Cephalosporin-3'-diazeniumdiolate
QSI: Group-sensing inhibitor
DGC: Diguanosine cyclase
PDE: Phosphodiesterase
PIA: Polysaccharide intercellular adhesion
